# Clinical application of 3D‐printed bolus in postmastectomy radiotherapy

**DOI:** 10.1002/acm2.70393

**Published:** 2025-11-28

**Authors:** Qianqian Liu, Xuming Chen, Zhekai Hu, Lingtong Hou, Yihang Zhang, Tiening Zhang, Shengyu Yao

**Affiliations:** ^1^ Department of Radiation Oncology Shanghai General Hospital Shanghai Jiao Tong University School of Medicine Shanghai P. R. China

**Keywords:** 3D‐printed bolus, Air gap, Postmastectomy, Radiation therapy

## Abstract

**Purpose:**

To evaluate the fitting accuracy and dose influence of 3D‐printed bolus (3DPB) during postmastectomy radiotherapy.

**Methods:**

Air gaps were assessed using cone‐beam computed tomography (CBCT) images for 27 breast cancer patients who received postmastectomy radiotherapy. Then a CIRS thoracic phantom was used to evaluate the dosimetric effects of different air gap sizes.

**Results:**

In CBCT images of patients, compared with conventional bolus (CB), 3DPB significantly reduced both the maximum and total air gap volumes by an average between 103.04 cm^3^ and 124.63 cm^3^, respectively. The average maximum gap height was 0.53 ± 0.20 cm for 3DPB and 2.23 ± 0.41 cm for CB (p<0.05). In the results as obtained from the phantom simulation, as gap height increased from 0.5 cm to 3.5 cm, D95 (dose received by 95% of the target volume) and D98 (dose received by 98% of the target volume) values for the superficial skin zone (SSZ), clinical target volume (CTV), and planning target volume (PTV) all decreased insignificantly. When the gap exceeded 2 cm, dose reductions became more pronounced in SSZ. Similar trends were observed in the CTV and PTV.

**Conclusions:**

For breast cancer radiotherapy patients immobilized using a vacuum bag, as evidenced by the CBCT images and phantom simulation, 3DPB provides a superior anatomical fit, reducing air gaps and associated dose loss in postmastectomy radiotherapy. When the air gap height ≤ 2 cm, dose attenuation with CB remains clinically acceptable, suggesting that 3D printing may not be necessary unless the skin in the gap area is at high risk for recurrence. However, for gaps > 2 cm, the dose reduction becomes significant, warranting the use of 3D‐printed boluses to ensure adequate target coverage.

## 1 INTRODUCTION

Breast cancer stands as a predominant cause of cancer‐related mortality among women globally. Adjuvant radiotherapy following breast cancer resection plays a crucial role in significantly reducing the risk of local recurrence.^1^, ^2^ Postmastectomy radiation therapy (PMRT) is essential in enhancing local control and overall survival for patients with high‐risk features.^3^ In radiotherapy, the dose to the superficial target area near the skin is often insufficient due to the combined effects of the build‐up effect and the thin chest wall in patients who have undergone radical mastectomy.^4^, ^5^ This insufficiency can compromise the efficacy of radiotherapy and may even increase the risk of local recurrence.^6^

Tissue‐equivalent bolus used in radiotherapy can significantly enhance the superficial skin dose, while also improving the uniformity of dose distribution.^7^, ^8^, ^9^ However, due to the irregular contours of the human body and the curvature of the chest wall, gaps often form between the conventional sheet bolus and the skin, especially in the axillary region, compromising the effectiveness of the treatment. 3D‐printed bolus (3DPB) can effectively reduce the size of gaps, thereby improving dose coverage on the skin surface and enhancing dose uniformity in the target.^10^, ^11^, ^12^ However, previous studies have primarily focused on comparisons based on planning CT images, without tracking the actual conditions throughout the treatment course.^13^, ^14^, ^15^ In practice, over the several weeks of radiotherapy, the size and location of air gaps can vary due to respiratory‐induced chest wall deformation, patient positioning changes, and bolus setup errors, all of which may lead to fluctuations in dose distribution.^16^, ^17^ While 3D printing workflows offer improved fit, they can significantly prolong treatment preparation times and incur additional medical costs. This study investigated the dosimetric impact of varying air gap sizes on target volumes and skin throughout the entire treatment period, aiming to identify the conditions under which the use of 3D‐printed boluses is warranted.

## 2 MATERIALS AND METHODS

### 2.1 Patient selection

The 27 cases of breast cancer who received radiotherapy after modified radical mastectomy in our hospital since January 2021 to May 2025 were selected. Of these, 12 cases used 3D‐printed bolus (3DPB) with 0.5 cm thickness and 15 cases used conventional bolus (CB) with 0.5 cm thickness. The patients were immobilized using vacuum‐lock bags in supine position with the head slightly tilted to the healthy side. The patients raised the ipsilateral upper limb while keeping the contralateral arm close to the body.

The process of 3D printing tissue compensators was as follows: 1) The patient's CT and virtual bolus data were imported into Mimics Research software to extract images and generate an STL file. 2) The stereolithography (STL) file was used in Materialise Magics software to design a mold for the bolus. 3) The mold data was printed using a UnionTech LITE600 printer with light‐curing resin. 4) Silicone was poured into the mold, cured for 24 h, and then removed to obtain a custom silicone tissue compensator.

The interval between these two CT simulations (prebolus and postbolus fabrication) was deliberately kept within 3 days for all cases. This short timeframe was critical to minimize potential changes in patient anatomy and to ensure the precise fit of the bolus over the course of the procedure.

For the conventional bolus (CB) group, a CT scan with CB bolus was first acquired as the planning CT. For the actual treatment delivery, the CB was physically placed on the patient's chest wall by radiation therapists. For the 3DPB group, a CT scan without bolus was first performed and then a 0.5 cm 3DPB was created according to the contour of the patient's chest wall. After placing the 3DPB, another CT scan was performed to acquire the planning CT image. All CT scans were performed on a Philips Brilliance BigBore™ (Philips Medical Systems, Madison, WI) CT, with a slice thickness of 0.25 cm and a voltage of 120 kV. All patients received 50 Gy in 25 fractions. The following inclusion and exclusion criteria were defined:

Inclusion criteria: 1) Female patients aged 18‐80 years; 2) Patients who underwent radical mastectomy with a postoperative pathological diagnosis of breast cancer; 3) Patients who received radiotherapy at our institution were treated using a conventional bolus or a 3D‐printed bolus (3DPB) during the course of their radiation therapy; 4) No evidence of distant metastatic disease on pretreatment systemic staging workup; 5) Availability of complete clinical data.

Exclusion criteria: 1) Female patients younger than 18 years or older than 80 years; 2) Patients who underwent breast‐conserving surgery for breast cancer; 3) Patients who only completed simulation/planning CT but did not complete the full radiotherapy course; 4) Patients with severe skin breakdown/ulceration on the chest wall during the treatment course.

### 2.2 Gap size comparison

During the treatment period, CBCT was performed once daily within the first 3 days after the start of treatment, followed by once weekly, resulting in a total of 6‐7 scans of each case. The gap was defined as a cavity between the bolus and the skin surface with CT values ranging from −800 to −1000 Hounsfield Unit (HU) in the CBCT images.^18^, ^19^ The vertical height and volume of the maximum gap and total volume of all the air gaps were measured. The vertical distance was measured as follows: the air gap was first automatically contoured based on CT value thresholds (−800 HU to −1000 HU), followed by a manual measurement of the maximum value of all distances from the skin surface normal direction to the inferior surface of the bolus within the contoured air gap, wherein the vertical height measurement was obtained by manually measuring the maximum surface‐normal distance within the gap. All gap statistics were confined to the skin‐to‐bolus interface within the planning target volume (PTV) area.

### 2.3 Phantom simulation

Due to the relatively poor image quality of CBCT, using it for dose calculation can lead to significant errors. Additionally, the length of CBCT was only 16 cm, which was insufficient to cover the entire treatment target area, making it unsuitable for evaluating the impact of air gaps on the dose distribution across the whole target. Therefore, we used a CIRS thoracic phantom to assess the dosimetric effects of different air gap sizes on the skin and target volume.

1) Contouring of structures

**FIGURE 1:**
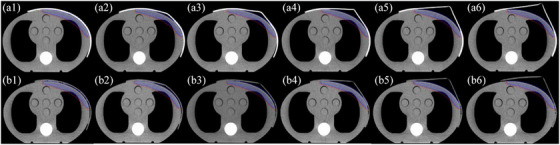
3D‐printed and conversional bolus with different sizes of air gaps (A: 3D‐printed bolus; B: conversional bolus; 1‐6:0‐3.5 cm); Clinical target volume (CTV): blue, planning target volume clinical (PTV): red, superficial skin zone (SSZ): yellow.

Both 3DPB and CB with a uniform thickness of 0.5 cm were used to cover the chest wall of the phantom for CT scan. Due to its density being close to that of air, expanded foam had minimal impact on radiation dose and was easy to cut, making it commonly used for patient positioning immobilization in radiotherapy. Therefore, this study utilized expanded foam to manually cut cones of varying heights, which were fixed between the phantom surface and the bolus to simulate the air gaps between the irregular human chest wall and the bolus. We artificially created gaps of different heights between the bolus and the skin (0.5 cm, 1 cm, 2 cm, 3 cm, 3.5 cm), as shown in Figure 1. CT scans were then performed for each gap size (0.0625 cm slice thickness). clinical target volume (CTV) was contoured as left radical mastectomy radiotherapy patients, anterior boundary included the entire skin surface; PTV was generated by expanding the CTV by 0.5 cm, with the anterior margin constrained to the skin surface. The superficial skin zone (SSZ) was defined as the area 0.3 cm below the skin surface within the PTV.

2) Dosimetric impact of gaps

**FIGURE 2:**
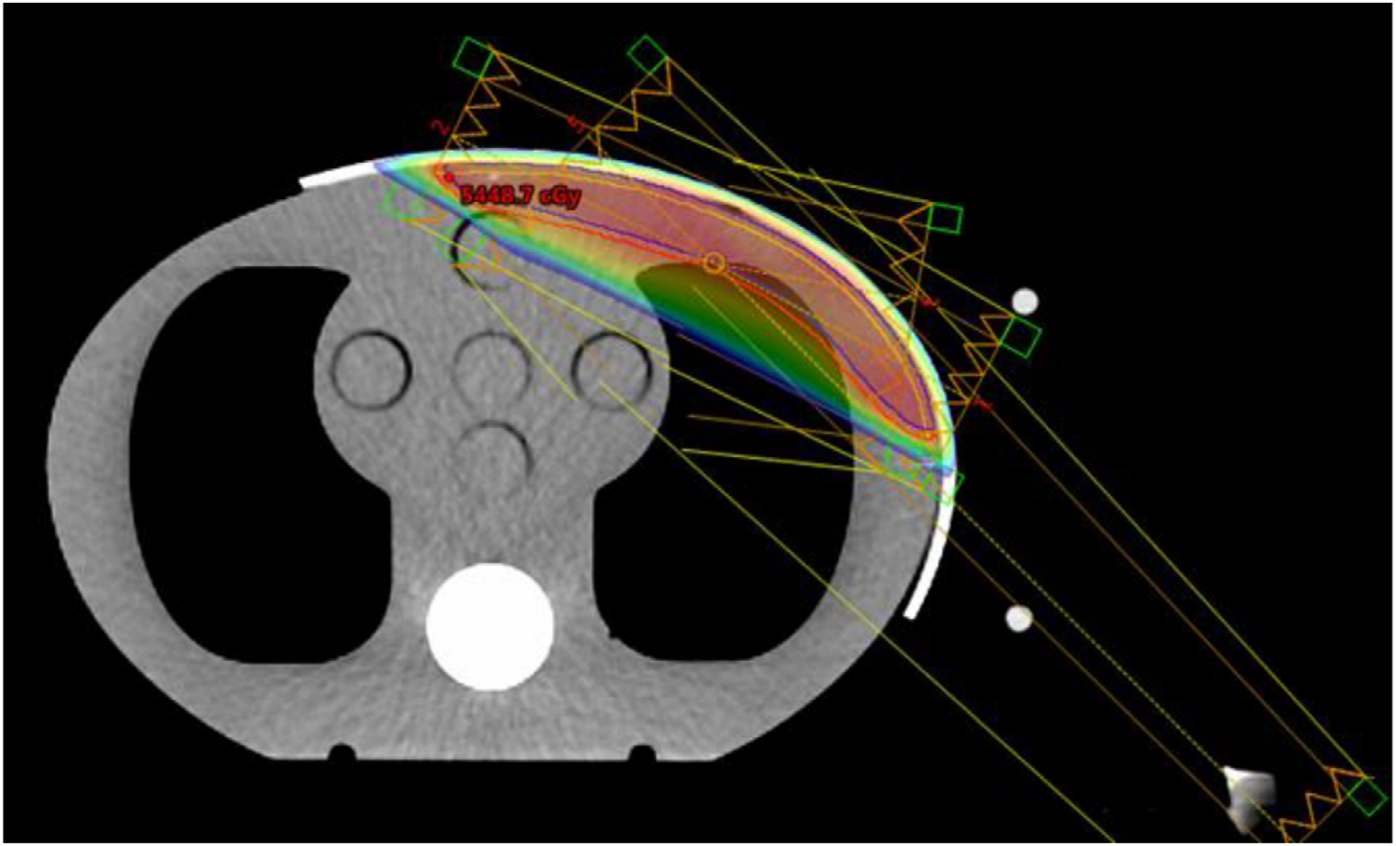
Beam arrangement diagram.

Intensity‐modulated radiotherapy (IMRT) plans were generated using a Varian Eclipse (13.6 Version) treatment planning system (TPS), modeled with the Linac iX (Varian, Palo Alto, CA, USA) linear accelerator. IMRT plans with a 6 MV photon beam using AXB algorithm contained two opposed tangential fields (118° and 296°) and the other three fields (100°, 135°, and 315°) that were used to control dose hotspots within the target volume (Figure 2). For the purpose of comparison, both plans with conventional bolus and 3D‐printed bolus were normalized to 95% of PTV covered by 50 Gy. Plans were designed on CT images with no air gap and then copied to other CT images with different gap sizes.

### 2.4 Statistical analysis

To assess the differences between the air gaps on CBCT and plans on phantom with different types of boluses, based on SPSS (V 22.0), independent‐samples t test was performed to assess the statistical equivalence between different types of boluses, where a *p* value of < 0.05 was considered statistically significant.

## 3 RESULTS

### 3.1 Gap sizes of the two types of boluses (clinical results)

**FIGURE 3:**
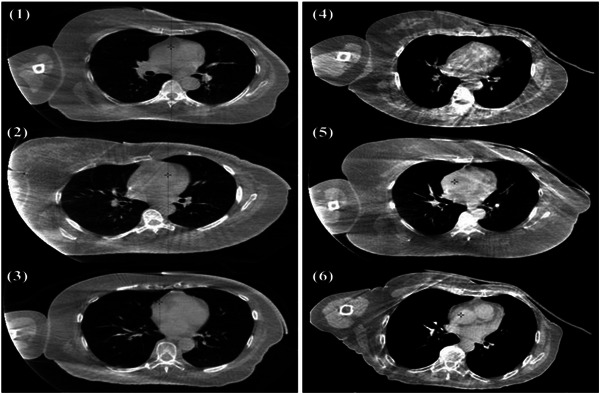
Cone‐beam computed tomography (CBCT) images of patients using conventional bonus (CB) and 3D‐printed bolus (3DPB) ((1–3): 3DPB, (4–6): CB).

A total of 176 CBCT images (98 for CB group and 76 for 3DPB group) were utilized in the gap size study. The images clearly demonstrated that the 3D‐printed bolus exhibited superior adhesion to the skin, with a significantly smaller air gap compared to the conventional bolus (Figure 3). The maximum air gap often occurred in the axillary region due to the anatomical concavity (3DPB‐Axillary: 80.3%, Anterior to the sternum: 17.1%, Other: 2.6%; CB‐Axillary: 86.7%, Anterior to the sternum: 10.2%, Other: 3.1%). In 3DPB group, one fraction with a gap size of 1.9 cm was excluded because the patient's arm moved significantly after the bolus was placed. A new CBCT scan was performed after adjustment.

**FIGURE 4:**
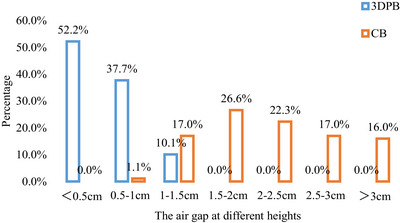
A bar chart illustrating the percentage distribution of different air gap heights, evaluated using different types of boluses.

The maximum gap height for 3DPB and CB was 1.3 cm and 3.7 cm, respectively with average values of (0.53 ± 0.20) cm and (2.23 ± 0.41) cm (p < 0.01). For 3DPB, gap heights smaller than 0.5 cm and 1 cm account for 52.2% and 89.9%, respectively, whereas for CB, the proportions were 0.0% and 1.1%, respectively. And 81.9% of the gaps in CB were larger than 1.5 cm and 55.3% were larger than 2 cm. The 3DPB markedly reduced the air gap height. The detail was shown in Figure 4.

Comparison of the maximum air gap volume and total air gap volume between the two types of boluses revealed superior performance by the 3DPB. The volumes for the 3DPB were (5.06 ± 4.64) cm^3^ and (7.56 ± 5.31) cm^3^, respectively, compared to (108.10 ± 53.86) cm^3^ and (132.19 ± 58.63) cm^3^ for the CB (p < 0.01). Detailed results are presented in Table 1.

**TABLE 1:** Comparison of gap sizes of two different boluses (X ± S).

Parameters	3D‐printed bolus	Conventional bolus	*T* value	*p* value
Maximum vertical height(cm)	0.53 ± 0.20	2.23 ± 0.41	−13.24	0.00
Maximum volume(cm^3^)	5.06 ± 4.64	108.10 ± 53.86	−6.58	0.00
Total volume(cm^3^)	7.56 ± 5.31	132.19 ± 58.63	−7.31	0.00

### 3.2 Dosimetric results

**FIGURE 5:**
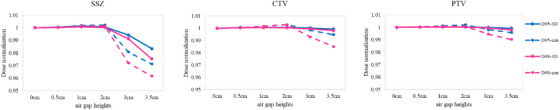
D95 and D98 of SSZ, CTV, and PTV vary with air gap height. 3D: 3D‐printed bolus, Con: Conventional bolus.

The D95 and D98 of the SSZ, CTV, and PTV decrease as the air gap increases. From 0.5 cm to 2 cm, the dose change was minimal. However, when the air gap exceeded 2 cm, the dose reduction increased significantly. At 3.5 cm, the D95 and D98 of the SSZ with the 3DPB decreased by 1.66% and 2.48%; the D95 and D98 of the CTV decreased by 0.07% and 0.19%; and the D95 and D98 of the PTV decreased by 0.07% and 0.19%, respectively. For the CB at a 3.5 cm air gap, the D95 and D98 of the SSZ decreased by 2.88% and 3.83%; those of the CTV decreased by 0.53% and 1.49%; and PTV decreased by 0.42% and 0.97%, respectively (Figure 5). When the air gap height was greater than 2 cm, the magnitude of dose reduction for the CB plan was significantly larger compared to the 3DPB plan.

## 4 DISCUSSION

The bolus is a commonly used as tissue‐equivalent material in tumor radiotherapy after radical mastectomy. It helps to compensate for the insufficient surface dose caused by high‐energy X‐rays. However, due to the natural curvature of the chest wall, especially the depression in the axillary region and the area of postsurgical hypertrophic scar tissue, CB often does not conform well to the body surface, which can result in large air gaps.^20^ These gaps may lead to insufficient surface dose and poor target dose uniformity, which may further reduce the effect of PMRT. In contrast, 3DPB is custom‐made based on the patient's surface contour, greatly improving the fit compared to CB.^10^, ^11^, ^12^, ^21^, ^22^ Wang J et al. assessed the conformability of the 3D‐printed bolus. They found the mean air gaps between the bolus and the chest wall in the center, upper outer, upper inner, lower outer, and lower inner quadrants were 0.04 cm (range: 0–0.33 cm), 0.18 cm (range: 0–0.68 cm), 0.04 cm (range: 0–0.36 cm), 0.04 cm (range: 0–0.26 cm), and 0.07 cm (range: 0–0.47 cm), respectively. These results demonstrated excellent conformability of the 3D‐printed bolus to the chest wall.^23^

However, previous studies were based on planning CT scans and did not track the actual situation during patient treatment. Ciaran Malone investigated the use of 3D‐printed bolus during real treatments for tumors in various body regions and found that for pelvic surface tumors, as much as 20% of 3D‐printed membranes were rated as having a “poor” fit (air gap > 1 cm), suggesting that actual treatment conditions may differ significantly from the planning CT.^24^ Our study found that for patients undergoing radiotherapy after radical mastectomy, even with the use of 3D‐printed bolus, 10.1% of treatment fractions had a maximum air gap > 1 cm. In one case, the air gap reached 1.9 cm due to the patient moving her arm after the bolus was placed, which increased the size of the gap. And the gaps during treatment were almost always larger than that in the planning CT. Therefore, the size of the gap can vary between treatment fractions due to changes in chest wall shape and bolus setup errors. This not only requires the professional skills and rigorous attitude of doctors and therapists, but also the development of more rational compensation methods and consideration of ways to minimize air gaps were particularly necessary. Ideally, image guidance should be performed for each treatment to ensure the gap size meets clinical standards. Due to the standardized geometry of conventional bolus, it often fails to achieve complete conformity with the irregular surface topology of the post‐mastectomy chest wall, particularly in regions with surgical scars or the concave contours of the axilla. And this gap cannot be eliminated by radiation therapists through positioning adjustments and the larger the chest wall depression, the greater the gap. In contrast, the 3D‐printed bolus is custom‐fabricated based on the individual patient's chest wall contour. Its patient‐specific geometry ensures a perfect match with the skin surface, including all inherent irregularities. The material possesses slight adhesiveness, promoting superior skin contact and facilitating therapists’ alignment using anatomical reference points, which substantially reduces setup‐related errors. And this highlights the advantage of 3D printed bolus (3DPB), which cannot be matched by manual positioning adjustments. From a subjective standpoint, both conventional bolus and 3D‐printed bolus require manual placement on the patient's chest wall by radiation therapists prior to each treatment session. Consequently, the magnitude of the air gap is highly dependent on the radiation therapist's positioning technique. To mitigate this variability, all radiation therapists at our institution undergo standardized training. The planning CT of two types of boluses and the CT for 3D‐printed bolus fabrication are acquired at a static phase of the respiratory cycle. However, respiratory motion during patient setup can lead to a changing air gap due to altered skin‐to‐bolus contact. Furthermore, pre‐treatment cone‐beam computed tomography (CBCT) imaging is routinely performed. If the CBCT reveals a significant air gap, the bolus is repositioned to minimize its separation from the skin surface, thereby reducing this potential source of manual error. The results of this study demonstrated that in CBCT images with CB, air gaps of less than 2 cm accounted for 44.7%, while gaps larger than 2 cm constituted 55.3%. In contrast, all air gaps observed in CBCT images with 3D‐printed bolus (3DPB) were under 2 cm. These results indicate that 3DPB significantly improved the conformity between the bolus and the chest wall by reducing air gaps in postmastectomy radiotherapy, which was consistent with previous research findings.

Studies have compared the impact of different air gap sizes on dose distribution. Martin et al. found that when the air gap was greater than 0.5 cm, the surface dose will be significantly reduced (about 10%).^25^ The previous results were observed that 3D‐printed boluses enhanced the precision of the dose delivered to the chest wall, achieving an accuracy within 3%. In stark contrast, the use of conventional step boluses led to a dose uncertainty of up to 6%.^26^ It was observed that the 3D‐printed bolus demonstrated exceptional adherence to the skin, with the mean air gap at the dosimetry point on the skin surface being as minimal as 0.1 cm.^27^ Takanen et al. showed that a personalized 3D‐printed bolus filled with ultrasound gel can readily replicate the consistency of a standard bolus, while providing precise coverage of the target area and ensuring tolerable acute and late toxicity levels.^28^ Butson et al. demonstrated that when air gaps were less than 1 cm, the reduction in skin dose remained within acceptable limits and over 90% of the maximum dose continued to be delivered across all skin areas.^29^ Chung et al. reported surface dose variations of up to 10% with a 1 cm air gap, using a 10 × 10 cm^2^ field size and a 60° incidence angle.^30^ Boman et al. observed an average surface dose reduction of 13.6% in Volumetric Modulated Arc Therapy (VMAT) due to the presence of a 1 cm air gap. However, these studies were conducted under fixed field sizes, using regular phantoms or virtual plans, and did not simulate the actual positions and sizes of air gaps during real patient treatments. We used a CIRS thoracic phantom to simulate the actual treatment conditions of postmastectomy breast cancer patients and studied the impact of varying air gap sizes on target dose and surface dose. The dose impact was not as significant as previously reported. For 3D‐printed bolus, even with a 2 cm air gap, the impact on surface dose parameters such as D95 and D98 remained negligible. In our CBCT images, the largest detected gap was only 1.9 cm. For conventional bolus, the largest observed gap was 3.5 cm, and the most significant dose reduction was in skin dose: D98 dropped by 3.8%. While, the air gap only affected part of the target area, not the entire region, so the dose reduction was localized.

Although 3DPB offers a dosimetric advantage, they can increase treatment waiting time and impose additional medical costs on the patient. These factors should be considered in clinical practice. Our study found that for a 2 cm gap in 3DPB, skin dose D95 and D98 decreased insignificantly; the D95 and D98 of PTV and CTV decreased, respectively‐virtually unchanged. For CB, a 2 cm air gap induced negligible dose variation. When the gap exceeded 2 cm, all dose metrics showed a clear trend of decline (Figure 5). The dose fall‐off was more pronounced in the CB plan compared to the 3DPB plan. Since the largest gaps are often found in the axillary region, it is advisable in clinical practice to first measure the maximum depth of the patient's axillary depression. If it ≤2 cm, CB may still be suitable, thereby improving treatment efficiency and reducing the patient's financial burden. However, if the depth clearly exceeds 2 cm, the use of 3DPB is recommended to effectively reduce the gap and improve target dose coverage.

Nevertheless, there are several notable shortcomings in this study. First, the number of cases in this study was insufficient. Further research should expand the number of clinical cases to enrich the data and thereby enhance the scientific rigor and reliability of the study results. Second, the CIRS phantom was used to simulate the gap of breast cancer patients' skin after fitting with bolus, which cannot completely replace the real gap between the surface and bolus. Third, this study was limited by the fact that only a planning study was conducted with no in vivo measurements performed. When conditions permit in the future, in vivo measurements should be performed to augment the dataset and corroborate the robustness of the theoretical study. And finally, the study's findings and conclusions are specifically applicable to setups utilizing vacuum‐bag immobilization systems. We should improve immobilization techniques emerge in the future, they are expected to simultaneously reduce setup errors and minimize gaps between the body surface and the bolus. Finally, future research will involve extending CBCT scans and generating synthetic CTs to investigate the actual treatment doses received by patients. We will continue to expand the number of clinical cases and follow up to explore the clinical outcomes of 3DPB.

## 5 CONCLUSIONS

Cone‐beam computed tomography (CBCT) was used to monitor the adherence of bolus in actual treatment and dose parameter calculations were performed for these gaps, and we found that the adhesion of the 3D‐printed bolus group was significantly better than that of the conventional bolus group. In the phantom experiment, 3DPB provides a superior anatomical fit, reducing air gaps and associated dose loss in postmastectomy radiotherapy, compared with CB. When the air gap height ≤ 2 cm, dose attenuation with CB remains clinically acceptable, suggesting that 3D printing may not be necessary to improve treatment efficiency and reducing the patient's financial burden unless the skin in the gap area is at high risk for recurrence. However, for gap > 2 cm, the dose reduction becomes significant, warranting the use of 3D‐printed boluses to ensure adequate target coverage.

## AUTHOR CONTRIBUTIONS

**Qianqian Liu**: Conceived and designed the analysis; contributed data or analysis tools; performed the analysis; wrote the paper.

**Xuming Chen**: Data organization and combing; Analyzed and interpreted the data; contributed to the writing of the paper.

**Zhekai Hu**: Data organization and combing; Analyzed and interpreted the data; contributed to the writing of the paper.

**Lingtong Hou**: Guided the analysis and interpretation of data; provided technical support for data management.

**Yihang Zhang**: Participated in data collection and analysis.

**Tiening Zhang**: Participated in data collection and analysis.

**Shengyu Yao**: Guided the analysis and interpretation of data; critically revised the manuscript for important intellectual content.

All authors read and approved the final manuscript.

## ETHICS APPROVAL AND CONSENT TO PARTICIPATE

This study was approved by the Institutional Review Board of Shanghai General Hospital, Shanghai Jiao Tong University School of Medicine, China (No.2022‐N‐09).

## CONFLICT OF INTEREST STATEMENT

The authors declare no conflict of interest.

## References

[1] Ragaz J , Jackson SM , Le N , et al. Adjuvant radiotherapy and chemotherapy in node‐positive premenopausal women with breast cancer. N Engl J Med. 1997;337(14):956‐962. 10.1056/NEJM199710023371402 9309100 10.1056/NEJM199710023371402

[2] Overgaard M , Nielsen HM , Overgaard J , et al. Is the benefit of postmastectomy irradiation limited to patients with four or more positive nodes, as recommended in international consensus reports? A subgroup analysis of the DBCG 82 b&c randomized trials. Radiother Oncol. 2007;82(3):247‐253. 10.1016/j.radonc.2007.02.001 17306393 10.1016/j.radonc.2007.02.001

[3] Overgaard M , Hansen PS , Overgaard J , et al. Postoperative radiotherapy in high‐risk premenopausal women with breast cancer who receive adjuvant chemotherapy. Danish Breast Cancer Cooperative Group 82b Trial. N Engl J Med. 1997;337(14):949‐955. Doi: doi: 10.1056/NEJM199710023371401 9395428 10.1056/NEJM199710023371401

[4] TUĞRUL T , EROĞUL O , Determination of initial electron parameters by means of Monte Carlo simulations for the Siemens Artiste Linac 6 MV photon beam. Rep Pract Oncol Radiother. 2019;24(4):331‐337. Doi: doi: 10.1016/j.rpor.2019.05.002 31193931 10.1016/j.rpor.2019.05.002 PMC6545364

[5] Dipasquale G , Poirier A , Sprunger Y , et al. Improving 3D‐printing of megavoltage X‐ray radiotherapy bolus with surface‐scanner. Radiat Oncol. 2018;13(1):203. 10.1186/s13014‐018‐1148‐1 30340612 10.1186/s13014-018-1148-1 PMC6194575

[6] Hou Y , Song Y , Sun X , et al. Multifunctional composite hydrogel bolus with combined self‐healing, antibacterial and adhesive functions for radiotherapy. J Mater Chem B. 2020;8(13):2627‐2635. 10.1039/c9tb02967b 32129372 10.1039/c9tb02967b

[7] EBCTCG McGaleP , Taylor C , et al. Effect of radiotherapy after mastectomy and axillary surgery on 10‐year recurrence and 20‐year breast cancer mortality: meta‐analysis of individual patient data for 8135 women in 22 randomised trials. Lancet. 2014;383(9935):2127‐2135. 10.1016/S0140‐6736(14)60488‐8 24656685 10.1016/S0140-6736(14)60488-8 PMC5015598

[8] Manger R , Paxton A , Cerviño L . Dosimetric assessment of brass mesh bolus for postmastectomy photon radiotherapy. J Appl Clin Med Phys. 2016;17(6):86‐96. 10.1120/jacmp.v17i6.6221 10.1120/jacmp.v17i6.6221 PMC5690501 27929484

[9] Dilson L , Challapalli S , Sourjya B , et al. Estimation of surface dose in the presence of unwanted air gaps under the bolus in postmastectomy radiation therapy: a phantom dosimetric study. Asian Pac J Cancer Prev. 2022;23(9):2973‐2981. 10.31557/APJCP.2022.23.9.2973 36172659 10.31557/APJCP.2022.23.9.2973 PMC9810302

[10] Lu Y , Song J , Yao X , et al. 3D printing polymer‐based bolus used for radiotherapy. Int J Bioprint. 2021;7(4):414. 10.18063/ijb.v7i4.414 34805595 10.18063/ijb.v7i4.414 PMC8600301

[11] Albantow C , Hargrave C , Brown A , et al. Comparison of 3D printed nose bolus to traditional wax bolus for cost‐effectiveness, volumetric accuracy and dosimetric effect. J Med Radiat Sci. 2020;67(1):54‐63. 10.1002/jmrs.378 32011102 10.1002/jmrs.378 PMC7063257

[12] Wang X , Wang X , Xiang Z , et al. The clinical application of 3D‐printed boluses in superficial tumor radiotherapy. Front Oncol. 2021;11:698773. 10.3389/fonc.2021.698773 34490095 10.3389/fonc.2021.698773 PMC8416990

[13] Zhang Y , Huang Y , Ding S , et al. A clinical trial to compare a 3D‐printed bolus with a conventional bolus with the aim of reducing cardiopulmonary exposure in postmastectomy patients with volumetric modulated arc therapy. Cancer Med. 2022;11(4):1037‐1047. 10.1002/cam4.4496 34939343 10.1002/cam4.4496 PMC8855922

[14] Wang DQ , Zhang N , Dong LH , et al. Dose‐volume predictors for radiation esophagitis in patients with breast cancer undergoing hypofractionated regional nodal radiation therapy. Int J Radiat Oncol Biol Phys. 2023;117(1):186‐197. 10.1016/j.ijrobp.2023.03.060 37001764 10.1016/j.ijrobp.2023.03.060

[15] Tian L , Mao R , Li D , et al. Superficial dosimetry study of the frequency ofbolus using in volumetric modulated arc therapy after modified radical mastectomy. Technol Cancer Res Treat. 2024;23:15330338241264848. 10.1177/15330338241264848 39129335 10.1177/15330338241264848 PMC11322943

[16] Boman E , Ojala J , Rossi M , et al. Monte Carlo investigation on the effect of air gap under bolus in post‐mastectomy radiotherapy. Phys Med. 2018;55:82‐87. 10.1016/j.ejmp.2018.10.023 30471824 10.1016/j.ejmp.2018.10.023

[17] Huang CY , Yang B , Lam WW , et al. Effects on skin dose from unwanted air gaps under bolus in an MR‐guided linear accelerator (MR‐linac) system. Phys Med Biol. 2021;66(6):065021. 10.1088/1361‐6560/abe837 33607641 10.1088/1361-6560/abe837

[18] Lamba R , McGahan J , Corwin M , et al. CT Hounsfield numbers of soft tissues on unenhanced abdominal CT scans: variability between two different manufacturers' MDCT scanners. AJR Am J Roentgenol. 2014;203(5):1013‐1020. 10.2214/AJR.12.10037 25341139 10.2214/AJR.12.10037 PMC5015439

[19] Glide‐Hurst C , Chen D , Zhong H , et al. Changes realized from extended bit‐depth and metal artifact reduction in CT. Med Phys. 2013;40(6):061711. 10.1118/1.4805102 23718590 10.1118/1.4805102

[20] Mayer HF . The use of a 3D simulator software and 3D printed biomodels to aid autologous breast reconstruction. Aesthetic Plast Surg. 2020;44(5):1396‐1402. 10.1007/s00266‐020‐01733‐y 32356154 10.1007/s00266-020-01733-y

[21] Park SY , Choi CH , Park JM , et al. A patient‐specific polylactic acid bolus made by a 3D printer for breast cancer radiation therapy. PLoS One. 2016;11(12):e0168063. 10.1371/journal.pone.0168063 27930717 10.1371/journal.pone.0168063 PMC5145239

[22] Robar JL , Moran K , Allan J . Intrapatient study comparing 3D printed bolus versus standard vinyl gel sheet bolus for postmastectomy chest wall radiation therapy. Pract Radiat Oncol. 2018;8(4):221‐229. 10.1016/j.prro.2017.12.008 29452866 10.1016/j.prro.2017.12.008

[23] Wang J , Xiang ZZ , Tan CF , et al. Individualized 3D‐printed bolus promotes precise postmastectomy radiotherapy in patients receiving breast reconstruction. Front Oncol. 2023;13:1239636. 10.3389/fonc.2023.1239636 38152364 10.3389/fonc.2023.1239636 PMC10751906

[24] Malone C , Gill E , Lott T , et al. Evaluation of the quality of fit of flexible bolus material created using 3D printing technology. J Appl Clin Med Phys. 2022;23(3):e13490. 10.1002/acm2.13490 35048501 10.1002/acm2.13490 PMC8906215

[25] Ciobanu AC , Petcu LC , Járai‐Szabó F , et al. Validation of a 3D printed bolus for radiotherapy: a proof‐of‐concept study. Biomed Phys Eng Express. 2025;11(2). 10.1088/2057‐1976/adb15d 10.1088/2057-1976/adb15d 39899900

[26] Park K , Park S , Jeon MJ , et al. Clinical application of 3D‐printed‐step‐bolus in post‐total‐mastectomy electron conformal therapy. Oncotarget. 2017;8(15):25660‐25668. 10.18632/oncotarget.12829 27784001 10.18632/oncotarget.12829 PMC5421959

[27] Wang X , Zhao J , Xiang Z , et al. 3D‐printed bolus ensures the precise postmastectomy chest wall radiation therapy for breast cancer. Front Oncol. 2022;12:964455. 10.3389/fonc.2022.964455 36119487 10.3389/fonc.2022.964455 PMC9478602

[28] Takanen S , Ianiro A , Pinnarò P , et al. A customized 3D‐printed bolus for high‐risk breast cancer with skin infiltration: a pilot study. Curr Oncol. 2024;31(9):5224‐5232. 10.3390/curroncol31090386 39330014 10.3390/curroncol31090386 PMC11431794

[29] Butson MJ , Cheung T , Yu P , et al. Effects on skin dose from unwanted air gaps under bolus in photon beam radiotherapy. Radiat Meas. 2000;32(3):201‐204. 10.1016/S1350‐4487(99)00276‐0

[30] Gray A , Oliver LD , Johnston PN . The accuracy of the pencil beam convolution and anisotropic analytical algorithms in predicting the dose effects due to attenuation from immobilization devices and large air gaps. Med Phys. 2009;36(7):3181‐3191. 10.1118/1.3147204 19673217 10.1118/1.3147204

